# Investigating Endogenous Hypercortisolism Prevalence in a U.S. Population With Resistant Hypertension: MOMENTUM Rationale and Design

**DOI:** 10.1016/j.jacadv.2026.102596

**Published:** 2026-02-16

**Authors:** Jorge Plutzky, Pam R. Taub, Deepak L. Bhatt, Omar Al Dhaybi, Vanita R. Aroda, Jan N. Basile, Michael J. Bloch, Matthew Budoff, Robert S. Busch, John B. Buse, Dianne Cheung, Elena A. Christofides, Ralph A. DeFronzo, Rodolfo J. Galindo, Yehuda Handelsman, Luke Laffin, John C. Parker, Florian Rader, Julio Rosenstock, Lance A. Sloan, Iulia Cristina Tudor, Daniel Einhorn

**Affiliations:** a Brigham and Women’s Hospital, Boston, Massachusetts, USA; b University of California-San Diego School of Medicine, La Jolla, California, USA; c Mount Sinai Fuster Heart Hospital and Icahn School of Medicine at Mount Sinai, New York, New York, USA; d Medical University of South Carolina, Charleston, South Carolina, USA; e Renown Institute for Heart and Vascular Health and University of Nevada Reno School of Medicine, Reno, Nevada, USA; f Lundquist Institute for Biomedical Innovation at Harbor UCLA Medical Center, West Carson, California, USA; g Albany Medical College, Albany, New York, USA; h University of North Carolina at Chapel Hill, Chapel Hill, North Carolina, USA; i University of California Los Angeles, Los Angeles, California, USA; j Endocrinology Associates, Columbus, Ohio, USA; k University of Texas Health Science Center, San Antonio, Texas, USA; l University of Miami Miller School of Medicine, Miami, Florida, USA; m The Metabolic Institute of America, Tarzana, California, USA; n Cleveland Clinic, Cleveland, Ohio, USA; o Accellacare Research/Wilmington Health, Wilmington, North Carolina, USA; p Cedars-Sinai Medical Center, Los Angeles, California, USA; q Velocity Clinical Research at Medical City Dallas, Dallas, Texas, USA; r Texas Institute for Kidney and Endocrine Disorders, Lufkin, Texas, USA; s Corcept Therapeutics Incorporated, Redwood City, California, USA

**Keywords:** cortisol, dexamethasone suppression test, endogenous hypercortisolism, prevalence, resistant hypertension

## Abstract

**Background:**

Hypertension is a leading modifiable risk factor for cardiovascular disease and premature death among adults. Up to 18% of individuals with hypertension have resistant hypertension (rHTN), which substantially increases the risk of adverse clinical outcomes. Endogenous hypercortisolism can result in rHTN through multiple mechanisms.

**Objectives:**

MOMENTUM (NCT06829537) is the first large, observational, multicenter study examining the prevalence of endogenous hypercortisolism among adults with rHTN in the United States.

**Methods:**

Target enrollment is approximately 1,000 participants. To be eligible, adults aged ≥18 years must have rHTN, defined using the American Heart Association criteria (systolic blood pressure ≥130 mm Hg despite ≥3 antihypertensive medications of different classes at maximally tolerated doses, including a diuretic, or ≥4 medications from different classes regardless of systolic blood pressure). Endogenous hypercortisolism is defined as cortisol level >1.8 μg/dL on the 1-mg overnight dexamethasone suppression test with adequate dexamethasone (≥140 ng/dL). The primary endpoint is endogenous hypercortisolism prevalence.

**Conclusions:**

MOMENTUM will provide new insight into endogenous hypercortisolism in patients with resistant hypertension.

Hypertension is a leading modifiable risk factor for cardiovascular disease, stroke, disability, and premature death, affecting nearly half of adults in the United States.^1^, ^2^, ^3^ In 2019, annual health care costs associated with hypertension care in the United States were $2,759 per person, totaling $219 billion annually nationwide.^4^ Lifestyle modifications in combination with blood pressure–lowering medications, the primary means of controlling hypertension, are effective in many patients.^3^ However, a significant percentage of individuals, estimated at 9% to 18%, exceed the American Heart Association (AHA) systolic blood pressure (SBP) target of ≥130 mm Hg despite using ≥3 different antihypertensive agents (including a diuretic) or require ≥4 medications from different classes to achieve control, a clinical condition defined as resistant hypertension (rHTN).^2^^,^^3^^,^^5^ Adults with rHTN have an increased risk of ischemic heart disease, myocardial infarction, transient ischemic attack, stroke, end-stage renal disease, and premature mortality,^2^^,^^3^^,^^6^^,^^7^ as well as greater health care resource utilization,^6^^,^^8^ compared with those with controlled hypertension. The high clinical and economic burden associated with rHTN underscores a need for improved diagnosis and management of this condition.^8^, ^9^, ^10^

Difficult-to-control hypertension despite lifestyle changes and optimized medical management may involve secondary contributing factors.^3^ Primary aldosteronism is one common—albeit widely underdiagnosed—cause of rHTN. Endogenous hypercortisolism is another known contributing rHTN cause which remains largely unstudied.^3^^,^^11^^,^^12^ Endogenous hypercortisolism is a state of chronic cortisol excess that can arise from several abnormalities, including production of excess adrenocorticotropic hormone (ACTH) from a pituitary or ectopic tumor or the autonomous secretion of cortisol in the adrenal gland.^13^^,^^14^ In individuals without the classic physical manifestations often associated with overt Cushing disease (an extreme form of hypercortisolism usually with a pituitary etiology), endogenous hypercortisolism presenting as hypertension often has an adrenal origin.^14^

Epidemiological studies in individuals with type 2 diabetes (T2D) and other comorbidities reveal an association between endogenous hypercortisolism and hypertension, including rHTN. The CATALYST study, the largest observational study of endogenous hypercortisolism prevalence, screened over 1,000 U.S. adults with difficult-to-control T2D and found that approximately one-quarter of these individuals had endogenous hypercortisolism. Furthermore, a significantly higher proportion of individuals with endogenous hypercortisolism had hypertension (89% vs 79%; *P* < 0.001) and were taking ≥3 antihypertensive medications (38% vs 23%; *P* < 0.001) compared to those without hypercortisolism.^15^ A prior meta-analysis of 6 studies in individuals with T2D reported that the presence of hypertension was associated with a 2-fold increase in the likelihood of endogenous hypercortisolism.^16^

The prevalence of endogenous hypercortisolism among adults with rHTN in the United States remains unknown. The standard overnight dexamethasone suppression test (DST)—involving a 1-mg dose of dexamethasone the night before a morning blood draw—is the most sensitive test for detecting adrenal hypercortisolism, the form most likely found in individuals presenting with T2D as their initial medical complaint.^13^^,^^17^^,^^18^ Despite the relative simplicity of this test, screening for endogenous hypercortisolism is uncommon due to the perceived rarity of the condition and the perception of screening challenges.^19^^,^^20^ The CATALYST study showed that by excluding common causes of false-positive results, the standard overnight DST can identify individuals with endogenous hypercortisolism, thus facilitating medical management.^15^^,^^21^ Furthermore, hypercortisolism was even more likely among participants on multiple antihypertensive agents, highlighting the need to consider hypercortisolism in those with rHTN, irrespective of their T2D status.^15^ Indeed, a 2012 study from Brazil found that 1 in 4 individuals with rHTN may have endogenous hypercortisolism.^22^ Here, we describe the rationale and design of the first large, observational, multicenter study assessing the prevalence of endogenous hypercortisolism in a population with rHTN (MOMENTUM; NCT06829537).

## Materials and methods

### Study design and participants

**Figure 1:**
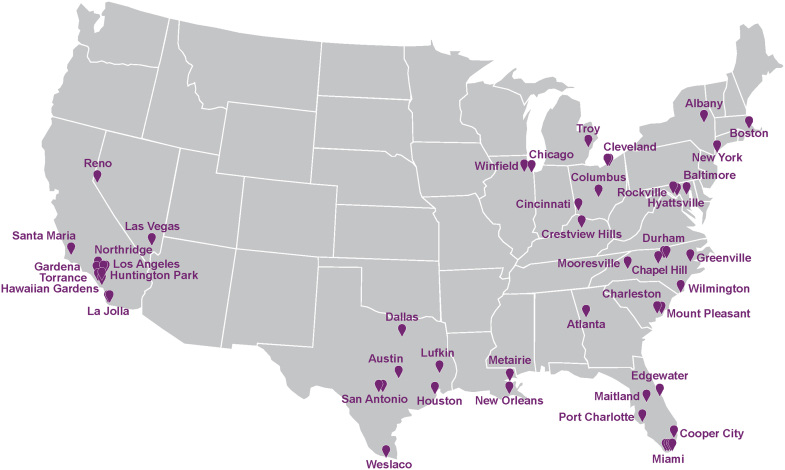
**MOMENTUM Study Sites^a^** The study is being conducted at approximately 52 sites reflecting a range of clinical practice settings and specialties. ^a^Multiple study sites in the same city are represented by separate pins.

MOMENTUM is an observational, noninterventional, multicenter study assessing the prevalence of endogenous hypercortisolism in approximately 1,000 individuals with rHTN (Central Illustration). Resistant hypertension is defined according to the 2017 AHA criteria,^2^^,^^3^ which were the U.S. guidelines available at the time of study commencement, as: 1) SBP above target (≥130 mm Hg) despite the concurrent use of 3 or more antihypertensive medications from different classes at maximally tolerated doses, with 1 medication being a diuretic; or 2) SBP at any level requiring the concurrent use of 4 or more antihypertensive medications from different classes. Clinically appropriate doses of antihypertensive medications are defined according to the investigator’s judgment and considered equivalent, for purposes of the study, to “maximally tolerated doses.” The study is being conducted at approximately 52 sites across the United States, reflecting a range of clinical practice settings and specialties, including academic, private practice, and research centers, with principal investigators drawn from cardiology, nephrology, primary care, and endocrinology (Figure 1). The study is funded by Corcept Therapeutics Incorporated, Redwood City, California.

**Central Illustration:**
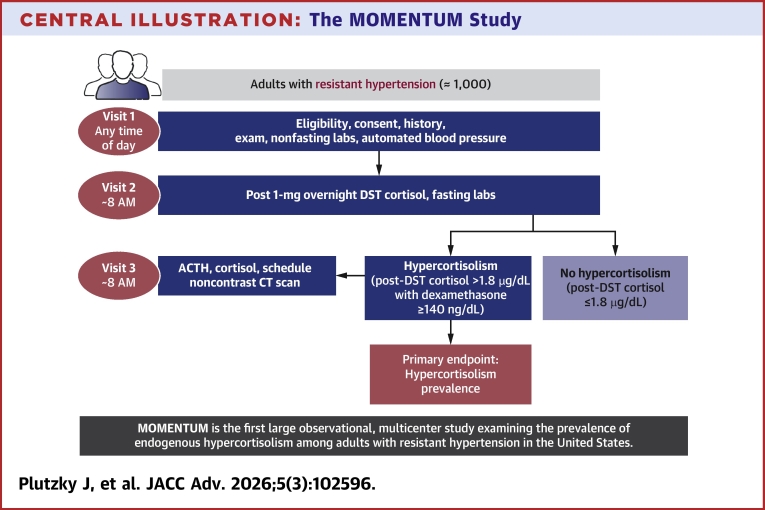
**The MOMENTUM Study** ACTH = adrenocorticotropic hormone; CT= computed tomography; DST = dexamethasone suppression test.

### Eligibility criteria

MOMENTUM is enrolling adults aged ≥18 years with rHTN. Exclusion criteria include factors that may result in an incorrect rHTN diagnosis and common causes of difficult-to-interpret (or “false-positive”) DST results. These include.

• Investigator-determined white coat hypertension or nonadherence to blood pressure medications
• Systemic glucocorticoid exposure ≤3 months before screening
• Estimated glomerular filtration rate of <30 mL/min/1.73 m^2^
• Severe acute psychiatric, medical, or surgical illness
• Excessive alcohol consumption
• Pregnancy or lactation
• Use of oral contraceptives
• Severe untreated sleep apnea

A complete list of exclusion criteria is shown in Table 1.

**Table 1:** MOMENTUM Study Inclusion and Exclusion Criteria

Inclusion criteria	• Aged ≥18 years • Resistant hypertension, defined according to AHA criteria (SBP ≥130 mm Hg despite the use of ≥3 antihypertensive medications of different classes, including a diuretic, at maximally tolerated doses or requiring ≥4 medications from different classes regardless of SBP)
Exclusion criteria	• Investigator-determined white coat hypertension (ie, elevated blood pressure in the office only) • Investigator-determined nonadherence to blood pressure medications • Systemic glucocorticoid medications exposure (excluding inhalers or topical) ≤3 months of screening • Historical eGFR <30 mL/min/1.73 m^2^ • Investigator-determined: ◦ Severe untreated sleep apnea ◦ Excessive alcohol consumption (eg, >14 units/week for male, >7 U/week for female) ◦ Severe acute psychiatric, medical, or surgical illness • Pregnant or lactating • History of congenital adrenal hyperplasia • Diagnosed with endogenous hypercortisolism and/or has used or plans to use endogenous hypercortisolism medications^a^ • History of hypersensitivity or severe reaction to dexamethasone • Individuals on OCPs who are unable to stop for ≥6 weeks prior to screening

AHA = American Heart Association; eGFR = estimated glomerular filtration rate; OCP = oral contraceptive pills; SBP = systolic blood pressure.

a Mifepristone, metyrapone, osilodrostat, ketoconazole, fluconazole, aminoglutethimide, etomidate, octreotide, lanreotide, pasireotide, or long-acting octreotide or pasireotide.

### Outcomes

The study’s primary endpoint is the prevalence of endogenous hypercortisolism in a population with rHTN. Endogenous hypercortisolism is defined as a cortisol level of >1.8 μg/dL after a 1-mg overnight DST with adequate concomitant dexamethasone levels (≥140 ng/dL). The dexamethasone level cutoff of 140 ng/dL, rather than the Endocrine Society–recommended cutoff of 220 ng/dL,^13^ is based on the normal range of the assay used in the central laboratory for the study (ARUP Laboratories). Dexamethasone is measured by a validated liquid chromatography with tandem mass spectrometry method with assay validation according to Clinical and Laboratory Standards Institute guidance under College of American Pathologists/Clinical Laboratory Improvement Amendments regulations. The threshold of 140 ng/dL corresponds to the lower reference limit of dexamethasone observed in the control population used to validate the assay and is concordant with reference intervals reported by other clinical reference laboratories. For participants with dexamethasone levels <140 ng/dL, the DST can be repeated using a 4-mg dose. If the dexamethasone level does not reach ≥140 ng/dL after repeat testing, the test is considered invalid, and the data are not included in the primary endpoint analysis.

Secondary endpoints include assessment of clinical and laboratory characteristics associated with an increased likelihood of endogenous hypercortisolism, proportion of individuals with markers of hyperaldosteronism and other causes of hypertension, and proportion of individuals with endogenous hypercortisolism and rHTN who have abnormal adrenal imaging. Exploratory endpoints include assessment of the proportion of participants with post-DST cortisol levels of 1.2 to 1.8 μg/dL and those with levels <1.2 μg/dL and their associated clinical and laboratory characteristics.

The study is also evaluating whether the degree of cortisol elevation post-DST is predictive of comorbidity severity or the presence and size of adrenal nodules. Several subgroup analyses are preplanned; patient demographics, clinical characteristics, blood pressure, medical history, and select medication use will be summarized overall and by endogenous hypercortisolism status within key subgroups. The subgroups will be defined by cardiac/carotid artery disorders (stratified by atrial fibrillation, heart failure, coronary artery disease, and other cardiac disorders), patients without cardiac or carotid artery disorders, broader cardiovascular disease status, renal function (estimated glomerular filtration rate ≥60, 45-59, and <45 mL/min/1.73 m^2^ using combined creatinine-cystatin C), and glycemic control (hemoglobin A1c categories ≥7.5%, ≥6.5%, 5.7%–<6.5%, and <5.7%). Simple and multiple logistic regression models will be used to evaluate factors associated with differences between groups.

### Procedures

**Figure 2:**
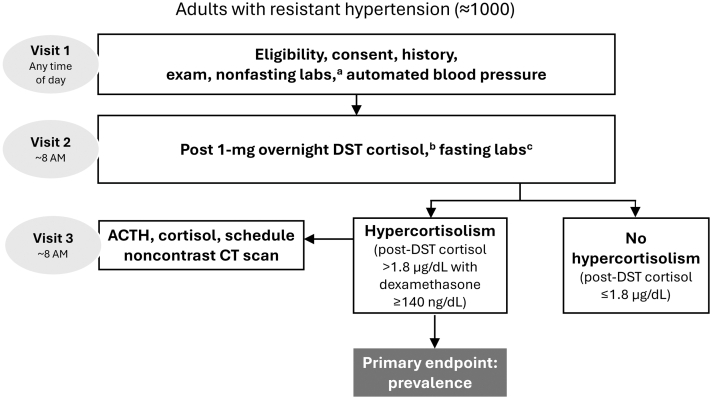
**Flow of Participants in the MOMENTUM Study** The study involves a total of 2 or 3 visits. At the initial screening visit assessing eligibility, participants will undergo an in-office automated blood pressure measurement and have a blood sample taken for nonfasting laboratory tests. All enrolled participants with resistant hypertension attend visit 2 having taken a 1-mg oral dose of dexamethasone the night before for hypercortisolism assessment (primary endpoint). Participants with a post-DST cortisol level of >1.8 μg/dL attend visit 3 for a nonfasted morning blood draw to test ACTH and cortisol levels and scheduling of a noncontrast adrenal CT scan. ^a^Plasma renin activity, aldosterone, dehydroepiandrosterone sulfate, N-terminal-pro-brain natriuretic peptide, hemoglobin A1c, fibrosis-4, aspartate aminotransferase-to-platelet-ratio index, uric acid, high-sensitivity C-reactive protein, complete blood count, comprehensive metabolic panel, estimated glomerular filtration rate, creatinine/cystatin-C, and urine albumin-to-creatinine ratio. ^b^The dexamethasone level must be ≥140 ng/dL for the DST to be valid. For participants with dexamethasone levels <140 ng/dL, the test may be repeated with 4 mg dexamethasone. ^c^ACTH, fasting glucose, and fasting lipids. ACTH = adrenocorticotropic hormone; CT = computed tomography; DST = dexamethasone suppression test.

The study involves a total of 2 or 3 visits (Figure 2). The initial visit 1 is a screening clinic appointment that may take place at any time of day. During this visit, participants provide written informed consent and are evaluated for study eligibility, including an in-office automated blood pressure measurement. SBP will be measured using the OMRON BP7450 (OMRON Healthcare, Inc) which automatically takes 3 measurements separated by 1 minute and provides the mean result. This device, which is supplied by the sponsor, will be used at each site, and site staff will be trained on its use. The clinical conditions for measuring blood pressure will follow the recommendations of the AHA and Centers for Disease Control and Prevention.^3^^,^^23^ Additional data collected at the initial visit include demographics, concurrent illnesses, history of cardiovascular diseases, and medical and medication history, including assessment of medication adherence. Physical measurements (height, weight, and waist circumference) are recorded, and a nonfasting blood sample is collected for laboratory assessments, including dehydroepiandrosterone sulfate, plasma renin activity, aldosterone levels, and a complete metabolic panel (Table 2). All enrolled participants with rHTN attend visit 2 within 2 weeks of visit 1, arriving at 8 AM (±1 hour) fasted for 8 or more hours and having taken a 1-mg oral dose of dexamethasone the night before (at 11 PM ±1 hour). Fasting blood samples are collected to assess cortisol, ACTH, glucose, and lipid levels (Table 2).

**Table 2:** Clinical Laboratory Variables Evaluated During the MOMENTUM Study by Clinic Visit

Visit 1: nonfasting; any time of day	
Hematology	Red blood cell count, distribution, and width; hematocrit; hemoglobin, mean corpuscular hemoglobin, mean corpuscular hemoglobin volume, and mean corpuscular volume; platelet count and mean platelet volume; white blood cell count; neutrophils;^a^ lymphocytes;^a^ monocytes;^a^ eosinophils;^a^ basophils^a^
Serum chemistry	Sodium, potassium, calcium, chloride, phosphorus, magnesium, creatinine, cystatin-C, creatine kinase, bilirubin (total and direct), albumin, alkaline phosphatase, lactate dehydrogenase, AST, ALT, blood urea nitrogen, uric acid, bicarbonate, total protein, eGFR_creatinine_, eGFR_cystatin-C_
Pituitary/adrenal hormone levels	DHEAS, plasma renin activity, aldosterone
Glucose control	HbA1c
Inflammatory markers	High-sensitivity C-reactive protein
Natriuretic peptide	NT-proBNP
Liver fibrosis	FIB-4, APRI
Urinalysis	UACR, urine pregnancy test for women of reproductive potential
Visit 2: fasting; 8 AM (dexamethasone taken prior evening)	
Pituitary/adrenal hormone levels	Serum cortisol, reflex dexamethasone levels for participants with post-DST cortisol >1.8 μg/dL, plasma ACTH
Glucose levels	Serum glucose
Lipid metabolism	Total cholesterol, low-density lipoprotein-cholesterol, high-density lipoprotein-cholesterol, very low-density lipoprotein-cholesterol, triglycerides
Visit 3: 8 AM (for subjects with post-DST cortisol >1.8 ug/dL)	
Pituitary/adrenal hormone levels	Plasma ACTH, serum cortisol

ACTH = adrenocorticotropic hormone; ALT = alanine aminotransferase; APRI = aspartate aminotransferase-to-platelet-ratio index; AST = aspartate aminotransferase; DHEAS = dehydroepiandrosterone sulfate; DST = dexamethasone suppression test; eGFR = estimated glomerular filtration; FIB-4 = fibrosis-4; HbA1c = hemoglobin A1c; NT-proBNP = N-terminal-pro-brain natriuretic peptide; UACR = urine albumin-to-creatinine ratio.

a Percent and absolute.

Participants with a post-DST cortisol level of ≤1.8 μg/dL are informed of their normal cortisol level, and no additional visits are required. Participants with a post-DST cortisol level of >1.8 μg/dL attend visit 3 within 2 weeks of the receipt of the DST result. Visit 3 entails a nonfasted morning blood draw to test ACTH and cortisol levels and scheduling of a noncontrast adrenal computed tomography scan. The total study duration is anticipated to be <4 weeks for most participants. Further clinical workup for participants with post-DST cortisol >1.8 μg/dL and potential treatment of the hypercortisolism is not mandated within the study. Rather, it will follow the local standard of care at each participating site.

Safety data (ie, adverse events, laboratory test results, and physical findings) are collected throughout the study, and patients are monitored on an ongoing basis. As this is a noninterventional study, an adverse event is defined as any unfavorable or unintended event related to a study procedure, such as a blood draw or imaging procedure, that occurs during the study.

### Statistical methods

The primary endpoint (prevalence of endogenous hypercortisolism in participants with rHTN) will be analyzed using descriptive statistics, with proportions and 95% CIs reported. All enrolled participants who meet the eligibility criteria and have a valid DST result will be included. Statistical analyses will use all available data without any imputation. Secondary endpoints will be summarized with descriptive statistics and 95% CIs. Clinical and laboratory findings will be evaluated for associations with endogenous hypercortisolism. Medical history will be coded using Medical Dictionary for Regulatory Activities v27 or later and presented by system organ class and preferred term. Descriptive statistics and 95% CIs will also characterize participants with markers of primary hyperaldosteronism (plasma renin activity and aldosterone) with and without endogenous hypercortisolism, those with primary hyperaldosteronism, endogenous hypercortisolism and rHTN, and those with endogenous hypercortisolism and rHTN with and without abnormal adrenal imaging. Continuous variables will be summarized by sample size (nonmissing), mean, SD, median, minimum, and maximum. Categorical variables will be summarized as numbers and percentages of participants in each category. Based on the CATALYST study,^15^ a sample size of approximately 1,000 participants is expected to provide a robust estimate of endogenous hypercortisolism prevalence, assuming a prevalence result in the range of 10 to 25%.

### Ethics and data sharing

The study is being conducted in accordance with Institutional Review Boards, local regulations, and ethical principles based on the Declaration of Helsinki and consistent with the International Conference on Harmonisation guidelines for good clinical practice. Participants must provide written informed consent before study-specific screening procedures. Participant confidentiality is maintained through multiple measures, including the use of unique participant identifiers on all study records. The study is funded and sponsored by Corcept Therapeutics Incorporated, Redwood City, California. The sponsor is responsible for the study design, trial oversight, data collection, and analysis. The statistical analysis plan will be prespecified and signed off before data analysis. The authors retained full editorial control and final responsibility for all content in this manuscript. No data are available for sharing at this time. On trial completion, deidentified data sets for the results from this trial may be made available to qualified researchers following submission of a methodologically sound proposal to datarequests@corcept.com. Data will be made available for such requests following the online publication of the primary results and for 1 year thereafter in compliance with applicable privacy laws, data protection, and requirements for consent and anonymization. Data will be provided by Corcept Therapeutics Incorporated.

## Discussion

Hypercortisolism is associated with adverse clinical consequences, particularly cardiovascular disease, as evident at any level of elevated cortisol.^24^, ^25^, ^26^, ^27^ In endogenous hypercortisolism, hypertension and thromboembolic events occur in up to 85% and 20%, respectively, of such individuals.^20^^,^^24^^,^^28^, ^29^, ^30^ Beyond vascular disease, endogenous hypercortisolism is associated with a broad spectrum of cardiometabolic complications, including insulin resistance, hyperglycemia, diabetes, fatty liver/metabolic dysfunction-associated steatotic liver disease, dyslipidemia, and visceral obesity, increasing the risk of cardiovascular morbidity and mortality.^20^^,^^29^^,^^31^ Individuals with endogenous hypercortisolism have an elevated risk of myocardial infarction and cardiac failure.^24^ Furthermore, cardiovascular disease is a leading cause of death in this population.^32^, ^33^, ^34^

The substantial burden of rHTN establishes an urgent need for greater understanding of its causes, which help inform new treatment strategies. Endogenous hypercortisolism can cause hypertension through direct and indirect mechanisms, including metabolic, vascular, renal, and cardiac action.^11^^,^^24^^,^^25^^,^^35^ In the renin-angiotensin system, excess cortisol induces increased hepatic synthesis of angiotensinogen, whereas renin may be low or suppressed, suggesting dysregulation of angiotensin receptor signaling. Although angiotensin II levels may be normal, the number of angiotensin II type 1 receptors is increased, resulting in enhanced response to angiotensin II and promoting vasoconstriction.^11^^,^^25^^,^^35^ In the vasoregulatory system, hypercortisolism may elevate levels of the vasoconstrictor endothelin-1 and impair the production of vasodilators, including prostacyclin and kallikreins, worsening endothelial dysfunction.^11^^,^^25^^,^^35^ Sustained increases in cortisol also downregulate nitric oxide synthase expression and inhibit nitric oxide signaling.^11^^,^^25^ In the sympathetic nervous system, excess cortisol enhances the pressor response to β-adrenergic agonists.^35^ Hypercortisolism increases sodium/hydrogen exchanger 3 activity in the proximal tubule, increasing renal sodium reabsorption, a main contributor to hypertension pathophysiology and the rationale for diuretic use in treating rHTN.^36^^,^^37^ Excess cortisol may exceed the capacity of 11β-hydroxysteroid dehydrogenase type 2 to convert cortisol to inactive cortisone, promoting higher cortisol levels and mineralocorticoid receptor activation.^11^^,^^35^^,^^38^ Notably, cortisol binds the mineralocorticoid receptor as avidly as aldosterone and can be present in 100- to 1,000-times higher plasma concentrations, expanding plasma volume through sodium retention.^11^^,^^38^

Other recognized secondary causes of rHTN include primary aldosteronism, renal parenchymal disease, and renal artery stenosis.^2^ Several of these, particularly aldosteronism, are part of the AHA guideline recommendations for routine screening in rHTN.^2^ European Society of Cardiology and Endocrine Society Clinical Practice guidelines recommend screening for primary aldosteronism in all adults with hypertension.^39^^,^^40^ Studies indicate up to 21% of patients with confirmed primary aldosteronism also have autonomous cortisol secretion.^31^^,^^41^ Although the AHA guidelines include endogenous hypercortisolism as a potential secondary cause of rHTN, routine screening is not recommended, reportedly due to lack of sufficient data.^2^

Few epidemiological studies have evaluated endogenous hypercortisolism in individuals with hypertension or rHTN. A 2025 systematic review identified 8 relevant studies published between 1977 and 2020, reporting endogenous hypercortisolism prevalence rates of 0% to 18.3% in individuals with hypertension.^42^ However, differences in testing approaches and definitions limited comparability, with only 1 study evaluating rHTN specifically. A 2012 study from Brazil involving 423 individuals reported that 27% of those with rHTN (defined by the same AHA criteria as in MOMENTUM) had endogenous hypercortisolism (based on 1-mg overnight DST).^22^ The CATALYST study demonstrated a 36.6% prevalence of endogenous hypercortisolism in individuals taking ≥3 blood pressure–lowering medications, although conducted in a population with T2D.^15^ To our knowledge, no study to date has investigated endogenous hypercortisolism in a U.S. population with rHTN.

MOMENTUM seeks to advance our understanding of the prevalence of endogenous hypercortisolism in a population with rHTN. These findings could support broader screening and more targeted treatment. Currently, diagnosis of endogenous hypercortisolism is often delayed for years.^19^^,^^20^^,^^43^ These delays may contribute to adverse clinical outcomes, as both the duration and the level of exposure to excess cortisol activity are significantly associated with increased morbidity and mortality risk.^19^^,^^20^^,^^26^^,^^43^, ^44^, ^45^ Although not assessed in this study, treatments for endogenous hypercortisolism are available, and more therapies are in development.^45^, ^46^, ^47^ In this context, MOMENTUM may help identify individuals with rHTN who could benefit from available and emerging therapies.

### Study limitations

Limitations of the study include that rHTN was determined based on a one-time in-office blood pressure measurement and investigator judgment as to participant adherence with at-home blood pressure checks and blood pressure medications. To address this limitation, in-office blood pressure was assessed in a consistent manner using the same in-office automated blood pressure device. Blood pressure was measured 3 times automatically, with a 1-minute interval between each measurement, without the investigator present. The mean blood pressure was used to determine eligibility. Out-of-office blood pressure measurements were reviewed with each participant to support the investigator in ensuring participants met the eligibility criteria. Similarly, adherence to blood pressure medications was based on participant interviews and investigator judgment. To minimize confounding of the DST results, the inclusion and exclusion criteria were carefully designed to rule out the most common confounding factors, including exogenous glucocorticoid exposure, oral contraceptive use, and specific comorbidities. Attainment of adequate dexamethasone levels was also ensured.

## Conclusions

The MOMENTUM study will provide key data on the prevalence and clinical characteristics of endogenous hypercortisolism in a large, representative population of adults with rHTN in the United States. These findings will help expand our understanding of the potential role of endogenous hypercortisolism as an important underlying secondary cause of rHTN, an area with currently limited data, and may also provide evidence to inform future clinical management guidelines for rHTN.

## Funding support and author disclosures

The study is funded by Corcept Therapeutics Incorporated, Redwood City, California. Dr Al Dhaybi discloses consulting fees from 10.13039/100004374Medtronic Inc. Dr Aroda discloses institutional contracts from 10.13039/100002429Amgen, Applied Therapeutics, 10.13039/100004325AstraZeneca, Biomea Fusion, 10.13039/100001003Boehringer Ingelheim, Corcept, Eli Lilly, Fractyl Health, 10.13039/501100004191Novo Nordisk, 10.13039/100004319Pfizer, Recordati, Rhythm Pharmaceuticals, 10.13039/100004339Sanofi, and Servier; she reports being a consultant to Fractyl Health, Mediflix, 10.13039/501100004191Novo Nordisk, and 10.13039/100004339Sanofi. Dr Basile discloses consulting fees from Alnylam/Roche, Blue Earth Diagnostics, Corcept, Eli-Lilly-SURPASS CVOT US National Leader, Idorsia-Hypertension, Mineralys, and Up to Date-HTN Section; and he is in the data safety monitoring board or advisory board of 10.13039/100004325AstraZeneca, Corcept, Idorsia, Mineralys Therapeutics, 10.13039/501100004191Novo Nordisk, and ReCor. Dr Bhatt discloses he is in advisory board of Angiowave, Antlia Bioscience, 10.13039/100004326Bayer, 10.13039/100001003Boehringer Ingelheim, CellProthera, Cereno Scientific, E-Star Biotech, High Enroll, Janssen, Level Ex, McKinsey, Medscape Cardiology, 10.13039/100004334Merck, NirvaMed, 10.13039/501100004191Novo Nordisk, Repair Biotechnologies, Stasys, and Tourmaline Bio; he is a member of board of directors of American Heart Association New York City, Angiowave (stock options), Bristol Myers Squibb (stock), DRS.LINQ (stock options), and High Enroll (stock); he reports being a consultant to Alnylam, Altimmune, Broadview Ventures, Corcept Therapeutics, Corsera, GlaxoSmithKline, Hims, SERB, SFJ, Summa Therapeutics, and Worldwide Clinical Trials; he is in the data monitoring committees of Acesion Pharma, Assistance Publique-Hôpitaux de Paris, Baim Institute for Clinical Research, Boston Scientific (Chair, PEITHO trial), Cleveland Clinic, Contego Medical (Chair, PERFORMANCE 2), Duke Clinical Research Institute, Mayo Clinic, Mount Sinai School of Medicine (for the ABILITY-DM trial, funded by Concept Medical; for ALLAY-HF, funded by Alleviant Medical), Novartis, Population Health Research Institute, and Rutgers University (for the NIH-funded MINT Trial); he received honoraria from American College of Cardiology (Senior Associate Editor, Clinical Trials and News, ACC.org; Chair, ACC Accreditation Oversight Committee), Arnold and Porter law firm (work related to Sanofi/Bristol Myers Squibb clopidogrel litigation), Baim Institute for Clinical Research (AEGIS-II executive committee funded by CSL Behring), Belvoir Publications (Editor in Chief, Harvard Heart Letter), Canadian Medical and Surgical Knowledge Translation Research Group (clinical trial steering committees), CSL Behring (AHA lecture), Duke Clinical Research Institute, Engage Health Media, HMP Global (Editor in Chief, Journal of Invasive Cardiology), Medtelligence/ReachMD (CME steering committees), MJH Life Sciences, Oakstone CME (Course Director, Comprehensive Review of Interventional Cardiology), Philips (Becker's Webinar on AI), Population Health Research Institute, WebMD (CME steering committees), and Wiley (steering committee); he is the deputy editor of Clinical Cardiology and Progress in Cardiovascular Diseases; he received patent from Sotagliflozin (named on a patent for sotagliflozin assigned to Brigham and Women's Hospital who assigned to Lexicon; neither he nor Brigham and Women's Hospital receive any income from this patent); he received research funding from Abbott, Acesion Pharma, Afimmune, Alnylam, Amarin, Amgen, AstraZeneca, Atricure, Bayer, Boehringer Ingelheim, Boston Scientific, CellProthera, Cereno Scientific, Chiesi, Cleerly, CSL Behring, Faraday Pharmaceuticals, Fractyl, Idorsia, Janssen, Javelin, Lexicon, Lilly, Medtronic, 10.13039/100004334Merck, MiRUS, Moderna, Novartis, Novo Nordisk, Pfizer, PhaseBio, Regeneron, Reid Hoffman Foundation, Roche, Sanofi, Stasys, and 89Bio; he received royalties from Elsevier (Editor, Braunwald’s Heart Disease); and he is the site co-investigator of Cleerly. Dr Bloch discloses grants or contracts from Amgen, Medtronic, Recor, and Sonivie and consulting fees from Coravie, Corcept, Idorsia, Medtronic, Recor, and Sonivie. Dr Budoff discloses grants or contracts and consulting fees from Corcept. Dr Busch discloses grant funding from Corcept; research funding from Eli Lilly, Novo Nordisk, and Novartis; he participates in speaker’s bureau for Amgen, AstraZeneca, Bayer, Eli Lilly, and Novo Nordisk. Dr Buse discloses grants or contracts from Corcept, Dexcom, GentiBio, and Novo Nordisk; he received consulting fees from Aardvark Therapeutics, Altimmune, Antag, Amgen, Aqua Medical, AstraZeneca, Boehringer Ingelheim, Corcept, Dexcom, Eli Lilly, embecta, GentiBio, Insulet, Metsera, Novo Nordisk, Tandem, Vertex, and Zealand; he reports payment for expert testimony from MiniMed and Novo Nordisk; support for meetings or travel from Boehringer Ingelheim, Corcept, Eli Lilly, Metsera, Novo Nordisk, Vertex, and Zealand; he is in the data safety monitoring board or advisory board of Altimmune, Antag, AstraZeneca, Corcept Therapeutics, Dexcom, Eli Lilly, Metsera, Novo Nordisk, Vertex, and Zealand; he holds the leadership or fiduciary role with American Diabetes Association; he received stock or stock options from Glyscend, Mellitus Health, Metsera, Pendulum Therapeutics, Praetego, and Stability Health; and he received receipt of equipment, materials, drugs, medical writing, gifts or other services from Corcept, Dexcom, GentiBio, Novo Nordisk. Dr Cheung discloses grants or contracts from Eli Lilly. Dr Christofides discloses grants or contracts from Ascendis, Corcept, Eli Lilly, Novo Nordisk, and Recordati; she received consulting fees from Chiesi, Crinetics, Adaptyx Bioscience, and Cristcot; she reports payment or honoraria from Abbott Nutrition, Ascendis, Bayer, Chiesi, Corcept, Crinetics, Eli Lilly, Recordati, and Xeris; she received support for meetings or travel from Corcept, Crinetics, and Recordati; she discloses patents from Adaptyx; she is in the data safety monitoring board or advisory board of Chiesi; and she holds the leadership or fiduciary role with ADA and MedCentral. Dr DeFronzo discloses grants or contracts from Eli Lilly, Boehringer Ingelheim, Corcept, and Regeneron; he received payment or honoraria from Corcept; and he is in the data safety monitoring board or advisory board of Corcept and Novo Nordisk. Dr Einhorn discloses he was employed by and owns stock in Corcept Therapeutics. Dr Galindo discloses grants or contracts from Boehringer, Dexcom, Eli Lilly, and Novo Nordisk; he received consulting fees from Abbott, AztraZeneca, Boehringer, Dexcom, Eli Lilly, Medtronic, and Novo Nordisk. Dr Handelsman discloses grants or contracts from Corcept, Ionis, Lilly, 10.13039/100004334Merck, Regeneron, and Verdiva Bio; he received consulting fees from Amgen, AstraZeneca, Bayer, Boehringer Ingelheim, Corcept, Regeneron, Sanofi, and Verdiva Bio; and he received payment or honoraria from Novo Nordisk. Dr Laffin discloses grants or contracts from Arrowhead Pharma, Crispr Therapeutics, Kardigan, Mineralys Therapeutics, and Retension; he received royalties or licenses from Elsevier; he reports consulting fees from Astrazeneca, Eli Lilly, Idorsia, Medtronic, Novartis, Recor, Ripple Medical, and Veradermics; he is in the data safety monitoring board or advisory board of Novo Nordisk; and he received stock or stock option from LucidAct Health. Dr Parker discloses consulting fees from Corcept, Novo Nordisk, and Insulet. Dr Plutzky discloses consulting fees from Amgen, Amarin, AstraZeneca, Bayer, Boehringer Ingelheim, CSL Behring, Corcept, Cleerly, Dexcom, Edwards, Esperion, Jazz, Lexicon, Lilly, Novo Nordisk, Novartis, Medtronic, Merck, and Roche. Dr Rader discloses consulting fees from Bristol Myers Squibb, Cytokinetics, Medtronic, ReCor Medical, AstraZeneca, Mineralys Therapeutics, and Idorsia. Dr Rosenstock discloses advisory panels from Applied Therapeutics, Boehringer Ingelheim, Eli Lilly, Hanmi, Intarcia, Novo Nordisk, Oramed, Sanofi, Scholar Rock, Structure Therapeutics, Terns Pharma, and Zealand; and he received research support from Applied Therapeutics, AstraZeneca, Boehringer Ingelheim, Eli Lilly, Novartis, Intarcia, Merck, Novo Nordisk, Oramed, Pfizer, and Sanofi. Dr Sloan discloses consulting fees from Boehringer Ingelheim, Corcept, Lilly Crinetics, Idorsia, Novo Nordisk, and Pfizer; he received honoraria from Bayer, Boehringer Ingelheim, Corcept, Madrigal, Pfizer, Sanofi, and Xeris; and he received research grants from Amgen, Boehringer Ingelheim, Corcept, Eli Lilly, Merck, and Novo Nordisk. Dr Taub discloses being a consultant to Amgen, Amarin, AstraZeneca, Bayer, Boehringer Ingelheim, CSL Behring, Dexcom, Edwards, Esperion, Jazz, Lexicon, Lilly, Novo Nordisk, Novartis, Medtronic, Merck, and Roche. Dr Tudor discloses being employed by and owning stock in Corcept Therapeutics.
